# Identification of Splicing Variation Associated with Parental Behavior in the Burying Beetles (*Nicrophorus orbicollis*)

**DOI:** 10.7150/jgen.123113

**Published:** 2025-10-20

**Authors:** Victoria P. Blythe, Xiangjia Min, S. Carmen Panaitof

**Affiliations:** Department of Chemical and Biological Sciences, Youngstown State University, 1 Tressel Way, Youngstown, OH 44555, USA.

**Keywords:** alternative splicing, transcriptome, parental care, burying beetle, *Nicrophorus orbicollis*

## Abstract

The molecular basis of parental behavior in burying beetles is not well understood. This study is a first attempt to evaluate the extent of alternative splicing (AS) as a source of transcriptome diversity that may facilitate flexible parenting behavior in this species. RNA-seq datasets from beetle parents exhibiting high or low care behavior and a newly available *Nicrophorus orbicollis* genome were used to perform AS analysis by AStalavista to comprehensively classify AS events, and specific patterns of splicing variation within and across parental groups were evaluated. Towards functional characterization, AS genes were annotated via protein family analysis. Transcriptome-wide AS profiles for each parental group were established, revealing no specific splicing patterns associated with either sex or parenting phenotype (high or low care behavior). Among simple types of AS events, intron retention was the most common (13%), while mutually exclusive exons were the least common (0.4%), with alternative acceptor (6%) and alternative donor (5%) occurring slightly more often than exon skipping (3%). Functional annotation highlighted AS genes belonging to protein families broadly linked to chemoreception, neuromodulation and JH function, all biological processes essential for the regulation of reproductive behavior and physiology. This analysis was successful in generating a large catalogue of AS events associated with parenting behavior in burying beetles. Additional analyses could expand upon this dataset to include tissue, development and species-specific splice variants, as well as functionally validate AS transcripts via RT-PCR to further clarify the role of functional AS in behavioral regulation in this species.

## Introduction

Burying beetles (Coleoptera: Silphidae: *Nicrophorus*) are a useful model to study the ecology and evolution of social behavior 1. *Nicrophorus orbicollis* male and female parents participate in similar behavioral tasks during breeding which include burying and preparing the reproductive resource, a small vertebrate carcass, into a carrion ball, and provisioning begging larvae with regurgitated carrion. Parental feedings are critical for larval survival 2 and post-hatching care provided by both parents results in larger broods being raised 3. Importantly, both males and females can provision offspring and successfully raise broods as single parents (uniparental care). The breeding cycle of *N. orbicollis* is marked by significant behavioral changes as parents coordinate tasks and adjust their response to cues from developing larvae 4. Our understanding of the genetic underpinnings of this behavioral flexibility is however limited. In recent years, several efforts relying on high-throughput sequencing methods 5-8 resulted in genome assemblies and a growing number of transcriptomic resources that have been used to explore genetic influences on parental behavior in *Nicrophorus spp*.

Our study is a first attempt to evaluate the extent of alternative splicing as a source of transcriptome diversity that may be linked to behavioral specialization for obligate care of young in *N. orbicollis* burying beetles. We hypothesized that behavioral flexibility during breeding and parental care will be underscored by flexible molecular responses reflected in variable, potentially sex- and/or care-level specific rates of splicing pre-mRNA processing likely to increase protein diversity and range of cellular functions under these conditions. We used the AStalavista 9 algorithm to systematically evaluate the transcriptome-wide alternative splicing profiles of male or female *N. orbicollis* in each parental group, highlighting the occurrence of several types of splicing variation (intron retention (IR), exon skipping (ES), alternative donor (AltD), alternative acceptor (AltA), mutually exclusive exons (MXE), and other complex events formed by two or more basic events). Genes found to be alternatively spliced were subject to further functional characterization, allowing us to identify alternative splicing loci associated with several protein families linked to the reproductive physiology and behavior of *N. orbicollis*. Evidence for an association between splicing patterns within each parental group and parenting phenotype (high or low care, male or female) was also evaluated, suggesting future work has the potential to clarify the role of functional alternative splicing as a genetic mechanism underscoring the regulation of behavioral variation associated with parental care in this species.

## Methods

### RNA-seq reads mapping and transcriptome assembly

The *N. orbicollis* genome (ASM1884563v2) with its corresponding annotation file and transcriptomic data from the same project (BioProject: PRJNA371654) of *N. orbicollis* adult male (N=20) and female (N=20) burying beetles were obtained from the National Centre for Biotechnology Information (NCBI) database. RNA-seq data were generated as part of a study 10 exploring differential gene expression associated with high or low care behavior (N=10 beetles in each parental group), representing high care females (HF), high care males (HM), low care females (LF) and low care males (LM). Tissue harvesting, behavioral data collection, RNA extraction and sequencing methods were described in the same study.

The *N. orbicollis* genome was indexed with Bowtie 2.2.6 11 and each RNA-seq dataset was mapped to the reference genome via TopHat 2.1.1 12. A GTF (gene transfer format) transcriptome assembly for each sample was generated in Cufflinks 2.2.1 13, using the BAM (binary alignment/map) file and reference GFF annotation file as input. To create a transcriptome assembly representative of the 40 RNA-seq datasets, all GTF assemblies from Cufflinks were merged via the Cuffmerge utility. Per group transcriptome assemblies were also generated with Cuffmerge, using the Cufflinks GTF assemblies from the 10 biological replicates in each parental group.

### Alternative splicing analysis

Alternating splicing (AS) events were detected within the merged transcriptome assembly for all samples and for each parental group using AStalavista 3.2 9. Both simple event types (ES, IR, AltD, AltA, MXE), and complex events (e.g., skipping of two or three exons in a row) 14 were detected. Conserved AS events between the parental groups were determined via comparison of the per parental group AStalavista results and were visualized with Venny 2.1 15.

### Functional annotation

The FASTA sequences for all transcripts within the merged transcriptome were acquired via the gtf_to_fasta utility in TopHat2. Using a custom database that included UniProtKB/Swiss-Prot and all *N. vespilloides* protein entries retrieved from NCBI, a BLASTX search was performed to find a single best hit for each FASTA sequence at a cutoff E-value ≤ 1e-5. A list of genes with their putative identities was subsequently generated via TargetIdentifier 16. ORFPredictor 17 was then used to identify predicted open reading frame (ORF) for all transcripts within the merged transcriptome. Predicted ORFs were next subject to rpsBLAST search against the Pfam database 18 to putatively identify the protein family encoded by each genomic locus.

## Results

### Identification of alternative splicing events

Across all 40 male and female *N. orbicollis* transcriptomes, ~1.4 billion reads (83.6%) successfully mapped to the reference genome. Of these, 8.7%, or approximately 122 million reads, represented multiple alignments, corresponding to reads which mapped to two or more genomic loci. The percentage of mapped reads was similar across beetle parental groups with an average of ~87% mapped reads in male transcriptomes and ~75% mapped reads in female transcriptomes. The merged GTF file from all *N. orbicollis* transcriptomes included a total of 76,897 unique transcripts.

AS analysis by AStalavista identified a total of 89,583 events within the merged transcriptome from the four parental groups. When considering individual types of AS, IR was revealed to be the most common (13%), while ES (3%) and MXE (0.4%) were the least common splice variants (Table 1). AltA and AltD splice variants were detected at intermediate levels (6% and 5%, respectively). With 65,083 (73%) entries detected across all four parental groups complex events represented other varieties of AS consisting of more than one basic AS event in *N. orbicollis* (Table 1). Each of the four merged parental transcriptomes included a range of 62,364 (HF) to 64,905 (HM) transcripts, with a relatively similar number of AS events uncovered in each parental group (52,840-53,788). Complex events, consisting of two or more basic AS events in a given splicing variation, accounted for a larger proportion of splice variants detected (65-66%), with the relative proportion of basic types of splice variants remaining remarkably consistent in each parental group (Table 1): IR (18%), AltA (6-7%), AltD (6%), ES (4%) and MXE (0.6%).

**Figure 1:**
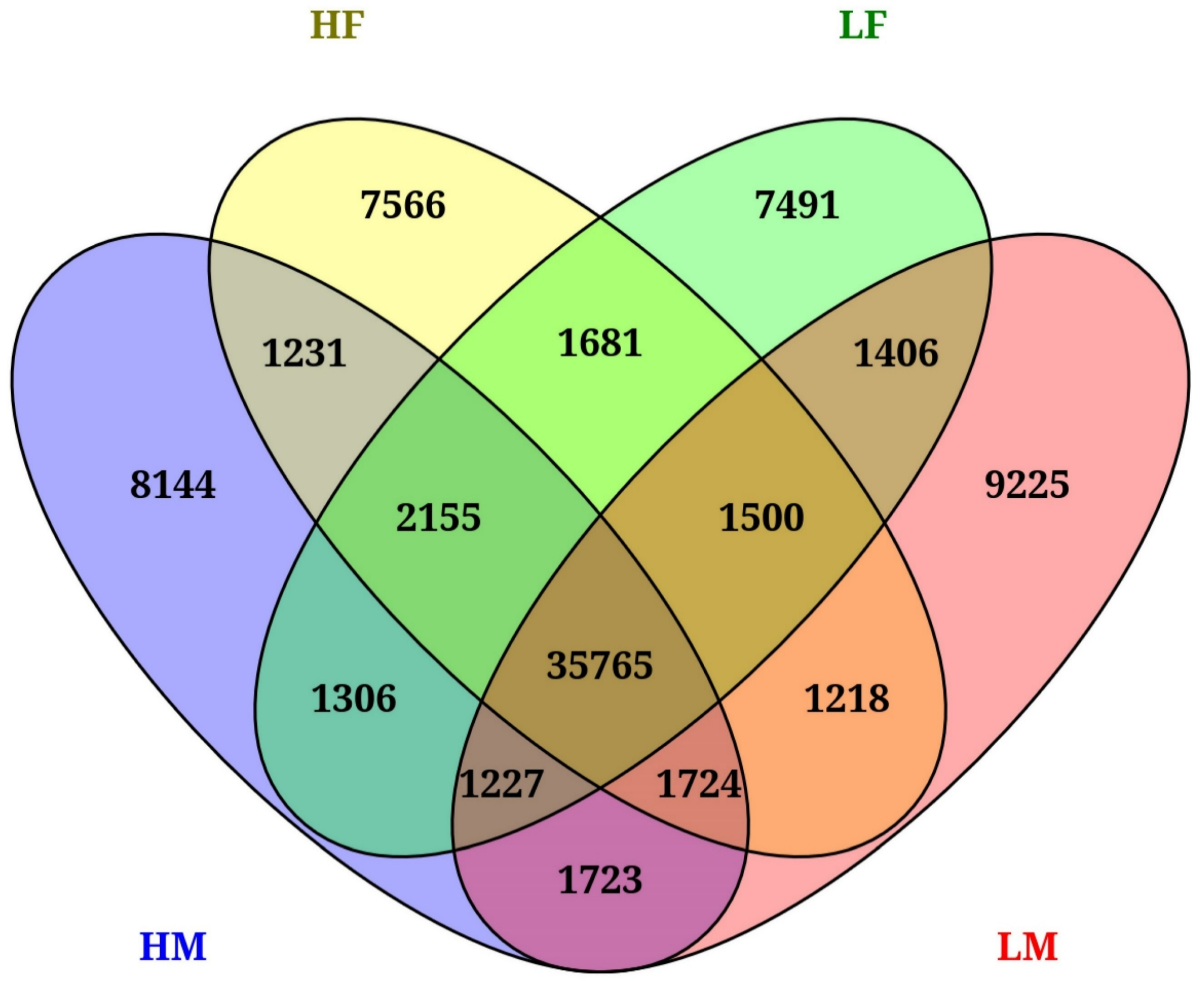
Conserved alternative splicing events shared by two or more *N. orbicollis* parental groups distinguished based on high care or low care parental behavior.

AS events unique to each parental group, as well as shared AS events across two or more parental groups, are highlighted in Figure 1. A relatively similar number of unique AS events were detected in each parental group (7,566 in HF, 8,144 in HM, 7,491 in LF and 9,225 in LM). We found a large overlap when comparing AS events across groups, with 50,936 events shared by two or more groups. High care (HF and HM) *N. orbicollis* parents exclusively shared 1,231 AS events while low care (LF and LM) parents shared 1,406, respectively. An impressive total of 35,765 AS events were shared by all four parental groups (Fig. 1).

### Functional annotation of transcripts

General descriptive features of the 76,897 unique transcripts identified based on the *N. orbicollis* RNA-seq dataset used in this study are summarized in Table 2. A total of 13,614 transcripts corresponded to unigenes (one transcript), while 10,785 of the 24,399 genomic loci (44.2%) were associated with more than one transcript. Functional annotation via BLASTX against the custom database revealed 57,659 matches (75%). ORF prediction yielded a total of 76,527 putative protein sequences (99.5%). Of the total predicted ORFs, 34,855 (45.5%) had a match in the Pfam database (Table 2). Only one Pfam annotation was utilized for genomic loci encoding multiple transcripts. Major protein families encoded by ≥10 genomic loci and the corresponding number of AS loci associated with each Pfam domain are listed in Supplementary Table S1.

Some of the highest numbers of AS loci were associated with general cellular functions and/or intra- and intercellular signaling (protein kinase, protein tyrosine kinase, trypsin, cytochrome p450, RNA recognition motif, 7 transmembrane receptor/rhodopsin family, ankyrin repeats, sugar (and other) transporter, short chain dehydrogenase, AMP-binding enzyme, ubiquitin carboxyl-terminal hydrolase, Ras family, mitochondrial carrier protein) (Table S1). We also uncovered a good representation of AS genes belonging to protein families expected to be strongly associated with various aspects of reproductive behavior and physiology in burying beetles (Table 3). Some of these AS loci belonged to protein families linked to chemosensory processing (7tm odorant receptor, 7tm chemosensory receptor, insect pheromone-binding family, pheromone biosynthesis activating neuropeptide). Interestingly, within families broadly linked to neuromodulation like pfam00001 (7tm_1), splice variants were identified for a number of dopamine and serotonin receptors (Table S1). For families related to Juvenile Hormone transport, action (vitellogenesis) and biosynthesis, we identified 16 AS loci for Haemolymph juvenile hormone binding protein, 3 AS loci for Vitellogenin_N/Lipoprotein amino terminal region and 1 AS locus for Hydroxymethylglutaryl-coenzyme A reductase (Table 3).

## Discussion

Post-transcriptional pre-mRNA processing via AS occurs in all eukaryotes and is a ubiquitous process in metazoans 19, with the largest proportion of alternatively spliced multi-exon genes found in *Homo sapiens* (~95% genes) 20 and a considerably lower frequency of AS encountered in invertebrates, including insects 21. Comparative studies in both invertebrate and vertebrate species have highlighted the importance of AS in generating transcriptome and proteome diversity, with novel, lineage-specific splice patterns thought to be driving molecular evolution and diversification of phenotypic traits 22.

Our current work is the first study to undertake a systematic analysis of the extent and specific event types of splicing pre-mRNA processing occurring in the burying beetles, *N. orbicollis*, an insect with an unusual reproductive pattern that includes extended parental care by both males and females. In our study that included 40 transcriptomes, ~44% of multi-exon genes expressed in parental *N. orbicollis* males and females produced 2 or more transcripts, a finding similar to reports from *Drosophila melanogaster* in which 31% genes are subject to AS 23. RNA-seq data genome mapping uncovered a similar number of transcripts and comparable AS profiles in *N. orbicollis* male and female parents, with no apparent sex-specific or caring phenotype-specific splicing patterns being detected. Using the AStalavista algorithm we were able to comprehensively classify AS event types in *N. orbicollis* and identify complex splicing patterns as the most prevalent type of splicing variation. When considering simple AS events separately, IR was the most common mechanism found in each of the four parental groups, a divergent feature compared to *D. melanogaster,* where ES appears to be more common than either IR or any of the other simple AS event types 24. Among eukaryotes, IR is more common in plants, and it is the dominant type of AS in fungi 19. When comparing AS profiles related to 12 metazoan genomes, including human among mammals, and fruitfly (*D. melanogaster*) and honeybee (*Apis mellifera*) among invertebrates, Sammeth et al. 14 uncovered a higher proportion of IR in the latter group, with the authors suggesting this AS event is favored by the shorter size of introns in invertebrates and the lower probability of protein disruption via a premature stop codon. In contrast, vertebrate species which included non-human primates (chimp), rodents (mouse, rat), chicken, frog and zebrafish registered a higher proportion of ES and complex events. Complex events were only slightly overrepresented in the fruitfly and honeybee, with an otherwise approximately equal abundance of ES, AltD, AltA, and IR event types 14.

The relatively large number of AS events (35,765) shared by all four parental groups would suggest a large proportion of splice variants may be underscoring the reproductive stage-specific expression of caregiving behavior, regardless of level of parental effort. These analyses would have benefitted from comparisons with splicing patterns of control, non-breeding *N. orbicollis* which were not collected as part of the published RNA-seq dataset we used. We would have similarly predicted a relatively large number of splice variants being exclusively shared by parents engaged in similar levels of parenting effort (i.e., HF and HM or LF and LM). Instead, we found a surprisingly large number of unique AS events to each parental group and a relatively low number of splicing variants exclusively shared by male and female parents in the same care level group which seems to further suggest that no specific splicing patterns can be associated with the intensity of parenting effort. Of note, our analysis identified transcripts and splice variants in male and female beetle heads, which contained brain and head fat body tissue, and thus the splicing patterns we uncovered can be attributed to only the tissue types therein. It would be useful to establish whether tissue- or developmental-stage specific splicing patterns exist in *N. orbicollis*, as has been uncovered in *Drosophila* 25, leading to a better understanding of how functional AS might be regulated in the burying beetles.

Protein family classification identified AS genes broadly linked to several functional categories expected to be strongly associated with the regulation of reproductive physiology and parental behavior of burying beetles such as chemosensory processing, neuromodulation/biogenic amine signaling, and Juvenile Hormone (JH) biosynthesis and action. Chemoreception is essential for carcass discovery, nestmate recognition and parent-offspring interaction 26, 27. Neuromodulation by biogenic amines signaling is equally expected to play a significant role in the regulation of flexible reproductive behaviors and parental care 28, 29. JH, a major reproductive hormone, has been repeatedly implicated in regulating the physiology of parental behavior of burying beetles 30. Our analysis represents the first transcriptome-wide cataloguing of splicing variation in *N. orbicollis*, highlighting transcriptomic diversity in actively parenting male and female burying beetles. Future work could build on these findings to include tissue- and developmental stage-specific analyses, as well as cross-species comparisons and functional validation by RT-PCR and should yield better insights into the role AS-related genetic variation may play in the regulation of the complex social behavior of burying beetles.

## Supplementary Material

Supplementary table.

## Figures and Tables

**Table 1:** Summary of alternative splicing events detected in male or female *N. orbicollis* parents distinguished based on high care or low care parental behavior.

			Alternative splicing events					
**Parental group**	Total transcripts	Total AS events	*ES*	*AltD*	*AltA*	*IR*	*MXE*	*Complex events*
High care female	62364	52840	2139 (4%)	2883 (6%)	3173 (6%)	9339 (18%)	304 (0.6%)	35002 (66%)
High care male	64905	53275	2185 (4%)	3062 (6%)	3512 (7%)	9536 (18%)	294 (0.6%)	34686 (65%)
Low care female	62415	52531	2138 (4%)	2915 (6%)	3203 (6%)	9425 (18%)	305 (0.6%)	34545 (66%)
Low care male	64339	53788	2168 (4%)	3027 (6%)	3371 (6%)	9596 (18%)	308 (0.6%)	35318 (66%)
All groups	76897	89583	2904 (3%)	4655 (5%)	5294 (6%)	11263 (13%)	384 (0.4%)	65083 (73%)

**Table 2:** Basic features of assembled *N. orbicollis* transcripts and their functional annotation.

Total genomic loci	24399
Loci having 1 transcript	13614 (55.8%)
Loci having more than 1 transcript	10785 (44.2%)
Total unique transcripts	76897
Average transcript length (bp)	2581
BLASTX match against custom database	57659 (75.0%)
Total predicted ORFs	76527 (99.5%)
Average ORF length (amino acids)	385
Total ORFs with a Pfam match	34855 (45.5%)

**Table 3:** Representative protein families for alternative splicing genes potentially associated with parental behavior and/or reproductive physiology in *N. orbicollis*

Pfam	Domain	Description	Total loci	AS loci
pfam00001	7tm_1	7 transmembrane receptor (rhodopsin family)	84	30
pfam06585	JHBP	Haemolymph juvenile hormone binding protein	48	16
pfam02949	7tm_6	7tm Odorant receptor	40	15
pfam00209	SNF	Sodium:neurotransmitter symporter family	23	9
pfam08395	7tm_7	7tm Chemosensory receptor	19	7
pfam02931	Neur_chan_LBD	Neurotransmitter-gated ion-channel ligand	17	8
pfam02932	Neur_chan_memb	Neurotransmitter-gated ion-channel	9	5
pfam01347	Vitellogenin_N	Lipoprotein amino terminal region	8	3
pfam03392	OS-D	Insect pheromone-binding family, A10/OS-D	7	2
pfam00003	7tm_3	7 transmembrane sweet-taste receptor of 3 GCPR	5	3
pfam05874	PBAN	Pheromone biosynthesis activating neuropeptide	5	1
pfam00351	Biopterin_H	Biopterin-dependent aromatic amino acid	3	2
pfam00368	HMG-CoA_red	Hydroxymethylglutaryl-coenzyme A reductase	3	1
pfam08473	VGCC_alpha2	Neuronal voltage-dependent calcium channel	2	2

## Abbreviations

AS: alternative splicing
*AltD*: alternative donor
*AltA*: alternative acceptor
*ES*: exon skipping
*IR*: intron retention
*MXE*: mutually exclusive exons
HF: high care females
HM: high care males
LF: low care females
LM: low care males
